# 136 Respiratory Infection Prevention in a Residential Treatment Facility for Pregnant and Parenting Persons with Substance Use Disorders

**DOI:** 10.1017/ash.2026.10436

**Published:** 2026-06-23

**Authors:** Cameron Fox, Haley King, Julia Donahue, Lindsay Daniels, Erin Brown

**Affiliations:** 1 UNC Health; 2 University of North Carolina Medical Center

## Abstract

**Background:** Intravenous penicillin G is the preferred first-line agent for intrapartum Group B streptococcus prophylaxis; however, many pregnant patients carry unverified penicillin allergy labels. Although approximately 10% of the general population reports a penicillin allergy, only about 1% have a true allergy, and these inaccurate labels often lead to unnecessary use of broader-spectrum antibiotics, highlighting the need for improved allergy verification and de-labeling in obstetric care. **Methods:** A retrospective chart review was conducted from January 2022 to December 2024 evaluating documented penicillin allergies in pregnant patients who received care and delivered within a state-wide health system. Patients were excluded if they had a penicillin allergy documented during or after the encounter, or if the allergy was identified as an erroneous entry. A study-specific allergy risk assessment was used to stratify patients into no allergy, low risk, moderate-to-high risk, and severe risk categories, using a conservative approach as the true severity of historical reactions could not be definitively verified through retrospective chart review alone. Patients categorized as no allergy were considered eligible for de-labeling. **Results:** Of 303 birth encounters screened, 285 met inclusion criteria; ten patients with previously removed penicillin allergy labels were excluded from the primary outcome analysis. Among the remaining included encounters, 51 patients (18.5%) were identified as eligible for penicillin allergy de-labeling based on chart review alone. Eligibility was driven by documentation consistent with non-allergic reactions, including intolerance symptoms (5.8%), history of prior penicillin tolerance (10.5%), and reported familial allergy without personal reaction history (2.2%). Referral patterns for allergy evaluation were assessed to identify additional de-labeling opportunities. Only 12 patients were referred to an allergist during pregnancy; however, 11 of these patients underwent penicillin challenge testing and were successfully de-labeled. The high success rate among referred patients highlights frequent mislabeling of penicillin allergies and suggests substantial missed opportunities for broader de-labeling in the obstetric population. **Conclusion:** Nearly one in five pregnant patients (18.5%) with documented penicillin allergies were eligible for de-labeling based on chart review alone. Low referral rates for allergy evaluation also indicate significant opportunities to expand penicillin allergy testing in obstetric care, further improving antibiotic selection and antimicrobial stewardship.

